# Epidemiology of rheumatic diseases in Mixtec and Chontal indigenous communities in Mexico: a cross-sectional community-based study

**DOI:** 10.1007/s10067-015-3148-y

**Published:** 2015-12-21

**Authors:** Flor Julián-Santiago, Conrado García-García, Imelda García-Olivera, María Victoria Goycochea-Robles, Ingris Pelaez-Ballestas

**Affiliations:** Sociomedical Sciences Program, Faculty of Medicine, National Autonomous University of Mexico (UNAM), Mexico City, Mexico; Rheumatology Department, General Hospital of México “Dr. Eduardo Liceaga”, Dr. Balmis 148. Colonia Doctores. Del. Cuauhtemoc., C.P. 06726 Mexico City, Mexico; Rheumatology Department, Hospital Regional de Alta Especialidad, Oaxaca, Mexico; Clinical Epidemiology Research Unit, Regional General Hospital No. 1 “Dr. Carlos McGregor Sánchez-Navarro”, Mexican Social Security Institute, Mexico. Sociomedical Sciences Program, Faculty of Medicine, UNAM, Mexico City, Mexico

**Keywords:** COPCORD, Indigenous population, Mexico, Oaxaca, Prevalence, Rheumatic diseases

## Abstract

This study aimed to estimate the prevalence of musculoskeletal (MSK) disorders and rheumatic diseases in the Chontal and Mixtec indigenous communities in the state of Oaxaca, Mexico, using the Community-Oriented Program for the Control of Rheumatic Diseases (COPCORD) methodology. After cross-culturally validating the COPCORD questionnaire for these communities, we conducted a cross-sectional, analytical, community-based census study using a house-to-house method. Positive cases of MSK disorders were assessed by primary care physicians and rheumatologists. The study population included participants aged ≥18 years from the indigenous communities of San Antonio Huitepec and San Carlos Yautepec. A total of 1061 persons participated in the study. Mean age was 46.9 years (standard deviation 19.9; age range 18–97 years); 642 (60.5 %) were women; 483 participants (45.5; 42.4–48.5 %) had MSK pain in the previous 7 days. Diagnoses were back pain 170 (16.0 %; 95 % confidence interval [CI] 13.8–18.3); osteoarthritis 157 (14.7 %; 95 % CI 12.7–17.0); rheumatic regional pain syndrome 53 (4.9 %; 95 % CI 3.7–6.4); rheumatoid arthritis 4 (0.3 %; 95 % CI 0.1–0.9); dermatomyositis 1 (0.09 %; 95 % CI 0.0–0.5); ankylosing spondylitis 1 (0.09 %; 95 % CI 0.0–0.5); systemic lupus erythematosus 1 (0.09 %; 95 % CI 0.02–0.5); and gout 1 (0.09 %; 95 % CI 0.0–0.5). 53.2 % had not received medical treatment for their disease. The prevalence of MSK disorders in indigenous communities in the Mixtec and Chontal regions is very high. The most common rheumatic diseases found were back pain and osteoarthritis. A high percentage of participants had not received medical care.

## Introduction

The global prevalence of musculoskeletal (MSK) disorders is estimated at over 20 %. The prevalence in different countries varies by diagnosis, ethnicity, age, and sex. However, these differences may arise from the methodologies used in different epidemiological studies [1–3]. The Community-Oriented Program for the Control of Rheumatic Diseases (COPCORD) grew out of an effort by the International League of Associations for Rheumatology (ILAR) and the World Health Organization (WHO) to collect information on the epidemiology of rheumatic diseases and their prevention in communities [4–6]. The methodology has been used in 26 countries including Mexico, where it was applied in a survey of five regions with different social, cultural, and economic backgrounds, yielding significant differences in the prevalence of MSK disorders and rheumatic diseases [7–12].

All studies concur that MSK disorders related to rheumatic diseases occur in at least 25 % of the population. Despite this high prevalence, rheumatic disorders are not considered a public health concern either locally or globally [13, 14]. Very few studies have attempted to explore the epidemiology of MSK disorders in indigenous populations [15, 16]. In Mexico, the government initiative to provide universal healthcare does not include rheumatic diseases. The health of indigenous populations in Mexico has been documented to be in decline owing to the limited availability of healthcare. This leads to the hypothesis that indigenous community dwellers with MSK disorders may be adversely affected by their remote location. However, the frequency of these disorders among indigenous peoples of Mexico is unknown [17].

Several Latin American countries, including Mexico, have initiated an effort to document the prevalence and impact of MSK disorders and rheumatic diseases on indigenous populations. This effort has been consolidated through the formation of the GLADERPO group (*Grupo Latino-Americano de estudio De Enfermedades Reumáticas en Pueblos Originarios* or Latin American Study Group on Rheumatic Diseases in Indigenous Peoples). As a first step in this effort, the COPCORD questionnaire was validated in several indigenous languages spoken by the native populations of Latin America [18].

About 10 % of the Mexican population is estimated to be of indigenous origin. Most communities are located in rural areas and must overcome major obstacles to access healthcare. One of Mexico’s 32 states, Oaxaca is located in the southeast and boasts the greatest cultural and linguistic diversity in the country, with 16 indigenous peoples recognized by the Declaration on the Rights of Indigenous Peoples [19, 20]. Our study aimed to estimate the prevalence of musculoskeletal disorders and rheumatic diseases in the Mixtec and Chontal indigenous communities in the state of Oaxaca, Mexico, by applying the COPCORD methodology.

## Material and methods

The present work was a cross-sectional, analytical, community-based COPCORD study design.

### Study population

In this study, a participant was considered indigenous if the following criteria were met: their language, self-identification, and ancestry are all recognized as indigenous, and they were residing on their ancestral territories [21]. This study focused on two indigenous communities, the Mixtec and Chontal, both from rural areas of Oaxaca. Both communities are governed in accordance with their customs and traditions (indigenous customary law). For example, the structure of both groups is a form of local organization built around the *cargo* system (also known as the civil-religious hierarchy), which is instrumental in helping these communities to make consensus-based decisions [22]. In addition to the *cargo* system, both groups also share certain socioeconomic conditions such as a high degree of marginalization and a high illiteracy rate (about 30 %). Where they differ is the access to health care, with the Mixteca community enjoying a health care coverage (the community is engaged in agriculture, livestock and trade; the first two activities predominate in the Chontal community).

#### Mixtec indigenous group

The Mixtecs are the fourth largest indigenous group in Mexico after the Nahuas, the Maya, and the Zapotecs. The Mixtec territory in Oaxaca covers an area of 40,000 km^2^ (around 15,500 mi^2^), encompassing a variety of microclimates and ecosystems. We administered the survey to the entire population of the San Antonio Huitepec community, located 85 km (52 mi) southwest of the state capital of Oaxaca; 70.6 % of those surveyed spoke Mixtec. The Mixtecs have the highest internal and external migration rates of all indigenous groups in Mexico, predominantly migrating to the USA [19, 21].

#### Chontal indigenous group

Owing to their geographical location, the Chontals from the municipality of San Carlos Yautepec are considered Highland Chontals, as opposed to the Lowland Chontals of the coastal region of Oaxaca. The territory of this community is located in the Sierra Sur region of the state where there is a hot and humid climate, and it occupies an area of 2,491.68 km^2^ (962 mi^2^). This community is geographically isolated and has a low migration rate [21].

### Sample selection

We surveyed indigenous subjects aged ≥18 years residing in the Mixtec area of the San Antonio Huitepec community and the Chontal area of the town of Santa Lucia Mecaltepec (San Carlos Yautepec). As a first step, we cross-culturally adapted and validated the COPCORD questionnaire to the native language of the target communities. The COPCORD’s Spanish questionnaire was translated and back-translated (orally and in writing) into the Mixteco and Chontal indigenous languages. The back-translation was verified in monolingual natives to validate semantic equivalence. During Stage 2, the questionnaire was administered to 100 subjects per indigenous community with the assistance of bilingual translators [18]. An updated census was used for the two participating communities.

The COPCORD questionnaire is designed to collect community data on pain and disability in joints and/or musculoskeletal soft tissues during the previous 7 days and/or any time in the past. In addition, the questionnaire evaluates treatment, use of complementary and alternative medicine, help-seeking behavior, and coping. Functional capacity was measured using a validated version of the Health Assessment Questionnaire Disability Index (HAQ-DI). The socioeconomic questionnaire included the measurement of such variables as education, marital status, monthly income, self-reported indigenous language proficiency, and healthcare type.

The COPCORD questionnaire was administered during house-to-house visits by previously trained bilingual interviewers using standardized techniques. When one or more participants were not located at the first visit, the household was visited on three more occasions at different times. If not located after three visits, the individual would be classified as reluctant to participate.

### Case definition

Respondents to the COPCORD questionnaire were considered positive when they reported recent MSK pain (in the previous 7 days) and/or historical pain. All cases were reviewed by primary care physicians trained in identifying rheumatic diseases. The medical examination was conducted on the same day and no more than 7 days after the interview by general practitioners in the community who received training on rheumatic diseases for 3 months before the study. Besides, one rheumatologist supervised and confirmed the diagnoses based on the international ACR criteria.

Cases of suspected rheumatic disease were assessed by a board-certified rheumatologist to confirm the diagnosis, using the American College of Rheumatology classification criteria for hand, hip, and knee osteoarthritis (OA) [23], rheumatoid arthritis (RA) [24, 25], fibromyalgia [26], and systemic lupus erythematosus (SLE) [27], as well as the Wallace criteria for gout [28], modified New York criteria for ankylosing spondylitis (AS) [29], and the Berlin criteria for inflammatory back pain [30]. Confirmed cases of rheumatic disease were referred to relevant public healthcare providers. Cases of non-rheumatic disease were diagnosed and referred to primary or specialized healthcare providers, as required. We do not have missing data in all questionnaire.

### Ethical aspects

This research project was approved by the Ethics Committee of the General Hospital of Mexico under code number DI/11/404D/05/123. The research project was presented at a community meeting in the target communities and was approved by the municipal authorities (in observance of the system of customs and traditions described above). All participants completed written informed consent before answering the questionnaire. When they did not know to read and write, they consent by placing their fingertip, which is legally accepted in Mexico.

### Statistical analysis

The prevalence and 95 % confidence interval of all variables were calculated. Continuous and ordinal, nominal, or categorical variables were reported as measures of central tendency and dispersion as percent distribution. The sociodemographic and clinical characteristics of the groups with and without rheumatic disease were compared using the *χ*^2^ test, Fisher’s exact test, or Student’s *t* test. A *p* value ≤ 0.05 was considered significant. The 95 % confidence interval was calculated where deemed relevant. Statistical analyses were performed using Stata version 12.0 for Windows [31].

## Results

The response rate in the surveyed communities was 100 %, with a total of 1,061 participants: 937 (88.3 %) from the Mixtec community and 124 (11.7 %) from the Chontal community. Table 1 shows the sociodemographic characteristics of the population as a whole.

**Table 1:** Demographic and socioeconomic characteristics of the study population (*n* = 1061)

Variable	*n* (%)
Gender (female)	642 (60.5)
Age (years); mean (SD)	46.9 (19.9)
Education level (years); mean (SD)	6.3 (4.7)
Married	775 (73.1)
Single	286 (26.9)
Unemployed	59 (5.5)
Indigenous community	
Mixtecs	937 (88.3)
Chontals	124 (11.7)
Native Language*, n* (%)	750 (70.6)
Monthly income in US dollars (year 2013)	
≤198.77	969 (91.3)
>198.77	92 (8.7)
Type of health care (*n* = 1061)	
None	40 (3.7)
IMSS-*Oportunidades*	679 (64.3)
National Health Insurance *Seguro Popular*	146 (13.7)
Full coverage	102 (9.6)
Private coverage	65 (6.1)
Other	29 (2.7)
Human waste management (*n* = 1051)	
Toilet bowl	721 (68.9)
Latrine	256 (24.1)
Other	74 (7.0)

*SD* standard deviation

Self-reported comorbidities were gastritis 237 (22.3 %), anxiety 206 (19.4 %), alcoholism 212 (19.9 %), depression 190 (17.9 %), obesity 183 (17.2 %), peripheral vascular disease 153 (14.4 %), blood hypertension 128 (11.9 %), and type 2 diabetes mellitus 100 (9.4 %).

**Fig. 1:**
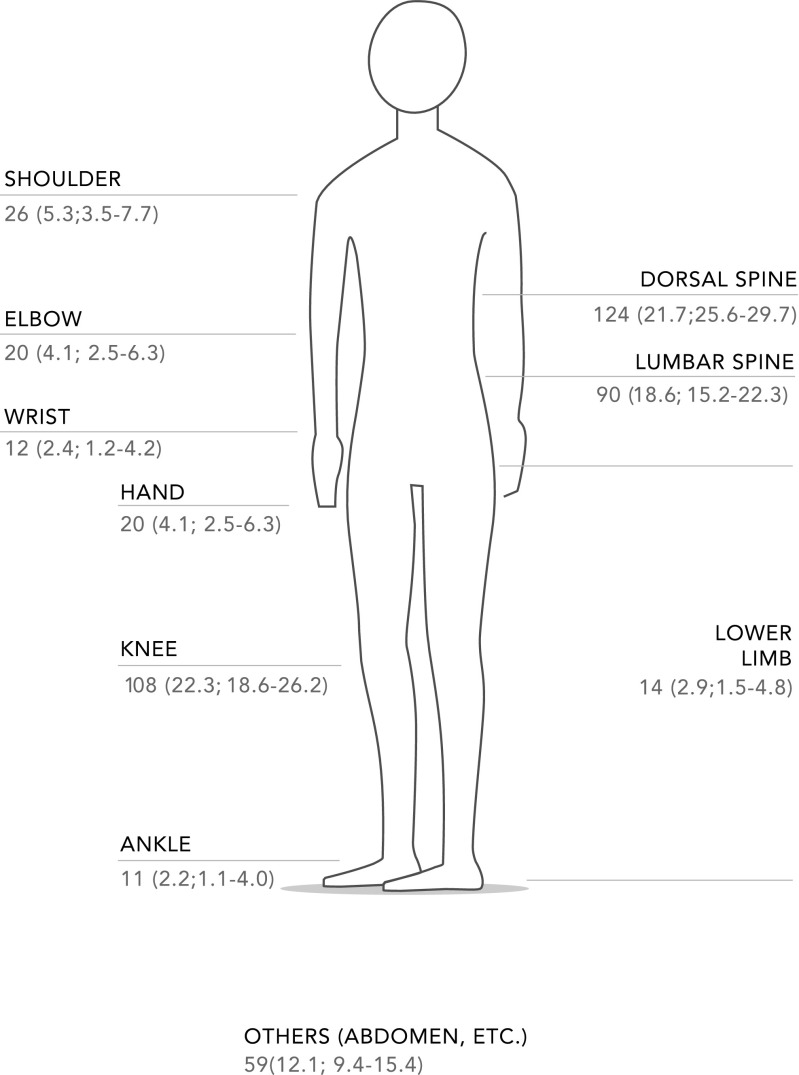
Musculoskeletal pain in the last seven days n (%; CI 95%)

Of the entire population surveyed, 483 participants (45.5 %; 95 % CI 42.4–48.5) reported having had MSK pain in the previous 7 days. In 376 (77.8 %) of these 483 participants, the pain was not associated with an injury. A total of 693 participants (65.3, 95 % CI 62.3–68.1) reported having had pain at some point in their lives. The pain was reported as the most severe in 29.8 % of participants during the previous 7 days, and in 27.2 % for historical pain. Forty-five percent of participants who reported pain in the previous 7 days and historical pain also reported taking some pain medication: 33.9 % took non-steroidal anti-inflammatory drugs, 13.7 % alternative or complementary medications, 2.9 % analgesics, and the rest took other drugs (corticosteroids, vitamin C, calcium, and various types of ointments). Only 0.2 % attended physical therapy and 2 % reported current limitations to performing daily activities. Table 2 compares these variables across the two participating indigenous populations. Figure 1 depicts the anatomical sites of reported MSK pain during the previous 7 days.

**Table 2:** Characteristics of pain and help-seeking care by indigenous group

Variable	Mixtec *n* = 937	Chontal *n* = 124	Total
Pain in the last 7 days (%; 95 % CI)	432 (46.1; 42.8–49.3)	51 (41.1; 32.3–50.3)	483 (45.5; 42.4–48.5)
Pain intensity			
*No pain*	4 (0.9)	–	4 (0.8)
*Some pain*	163 (37.7)	16 (31.3)	179 (37.0)
*Severe pain*	143 (33.1)	13 (25.4)	156 (32.2)
*The most severe pain*	122 (28.2)	22 (15.6)	144 (29.8)
Historic pain	612 (65.2; 62.0–68.2)	81 (65.3; 56.2–73.6)	693 (65.3; 62.3–68.1)
Functional limitation in the last 7 days, *n* (%)	*n* = 431^a^	*n* = 51	*n* = 482 ^a^
No limitation	385 (89.3)	44 (86.2)	429 (89)
Past limitation	35 (8.1)	7 (13.7)	42 (8.7)
Current limitation	11 (2.8)	0	11 (2.2)
Functional capacity, HAQ-DI score^b^	0 (0–0.2)	0 (0–0.2)	0 (0–0.2)
Treatment for pain in the last 7 days, *n* (%)	199/432 (46.0)	19/51 (37.2)	218/483 (45.1)
Treatment for historic pain, *n* (%)	228/611 (37.3)	30/81 (37.0)	318 /693 (45.8)
Type of health care professional			
Non-homeopathic	177 /432 (40.9)	16/51 (31.3)	193/483 (39.9)
Traditional alternative medicine^c^	16/432 (3.7)	0	15/483 (3.1)
Self-care	15/432 (3.4)	3/51 (5.8)	18 /483 (3.7)
Not seeking care	225/432 (52.0)	32/51 (62.7)	257/483 (53.2)
Coping with discomfort	*n* = 313 ^a^	*n* = 21 ^a^	*n* = 334 ^a^
No coping	8 (2.5)	2 (9.5)	10 (2.9)
Some coping*	73 (23.3)	12 (57.1)	85 (25.4)
Good coping*	232 (74.1)	7 (33.3)	239 (71.5)

*NS* not significant, Chi-square test (dichotomous). *One-way analysis-of-variance* (ANOVA), *SD* standard deviation, *IQR* interquartile range

*Significant at *α* = 0.05

^a^Missing data

^b^Median (IQR) median (IQR); Kruskal-Wallis test

^c^Spiritualist, bone setter, herbalist, traditional healer, or chiropractor

### Prevalence of MSK and rheumatic diseases

Table 3 shows overall results of the prevalence of confirmed MSK and rheumatic diseases in subjects with positive MSK pain, and provides a comparison across the groups. The most prevalent diseases were mechanical back pain 170 (16 %; 95 % CI 13.8–18.3), OA 157 (14.7 %; 95 % CI 12.7–17.0), and rheumatic regional pain syndrome 65 (6.1 %; 95 % CI 4.7–7.7), inflammatory back pain 44 (4.4 %; 95 % CI 3.0-5.5). The overall prevalence of RA was 4 (0.3 %; 95 % CI 0.1–0.9). Significant differences were observed in the prevalence of MSK diseases across the study groups.

**Table 3:** Prevalence of musculoskeletal diseases

MSK diseases	Total *n* = 1061 (%; IC 95 %)	Mixtecs *n* = 937 (%; IC 95 %)	Chontals *n* = 124 (%; IC 95 %)
Mechanical back pain	170 (16.0; 13.8–18.3)	157 (16.7; 14.4–19.3)	13 (10.4; 5.7–17.2)^a^
Inflammatory back pain ^b^	44 (4.1; 3.0–5.5)	36 (3.8; 2.7–5.2)	7 (5.6; 2.2–11.2)
Osteoarthritis ^c^	157 (14.7; 12.7–17.0)	117 (12.5; 10.4–14.7)	40 (32.2; 24.1–41.2) ^a^
MSK disorders	65 (6.1; 4.7–7.7)	64 (6.8; 5.3–8.6)	1 (0.8; 0.02–4.4) ^a^
Rheumatic regional pain syndrome	53 (4.9; 3.7–6.4)	39 (4.1; 2.9–5.6)	14 (11.2; 6.3–18.2) ^a^
Rheumatoid arthritis	4 (0.3; 0.1–0.9)	4 (0.4; 0.1–1.0)	–
Systemic lupus erythematosus	1 (0.09; 0.002–0.5)	1 (0.1; 0.002–0.5)	–
Dermatomyositis	1 (0.09; 0.002–0.5)	1 (0.1; 0.002–0.5)	–
Ankylosing Spondylitis	1 (0.09; 0.002–0.5)	1 (0.1; 0.002–0.5)	–
Gout	1 (0.09; 0.002–0.5)	1 (0.1; 0.002–0.5)	–
Family with multiple cases	1 (0.09; 0.002–0.5)	1 (0.1; 0.002–0.5)	–

^a^ *p* < 0.01

^b^Back pain Questionnaire was administered to 187 respondents, of which 132 met at least one criterion of 5 selected for the questionnaire. The most common criterion was improvement in pain level with exercise (*n* = 124), followed by morning stiffness >30 min (*n* = 93), night pain (*n* = 44), buttock pain (*n* = 36)

^c^Localized and generalized OA

A significant association was found between rheumatic diseases and older age; lower education level; and the presence of comorbidities such as blood hypertension, gastritis, anxiety, and depression (*p*-value <0.05) (Table 4).

**Table 4:** Association between socio-demographic and clinical variables with the presence or absence of a diagnosed MSK diseases (*n* = 1061)

	Without MSK diseases *n* = 677; *n* (%)	MSK diseases *n* = 384; *n* (%)	*p*
Female gender	423 (62.4)	219 (57.2)	0.095
Mean age, mean (SD)a	48.8 (19.8)	43.5 (19.4)	<0.001
Marital status (married)	507 (75.0)	269 (69.9)	0.052
Mean years of formal education, mean (SD)	6.4 (4.6)	7.4 (4.7)	<0.001
Blood hypertension	102 (15.0)	26 (6.7)	0.003
Gastritis	173 (25.5)	64 (16.6)	0.006
Anxiety	153 (22.6)	53 (13.8)	<0.001
Depression	149 (22.1)	41 (10.9)	<0.001
Smoking	18 (2.6)	21 (5.2)	0.03
Indigenous language	484 (82.3)	266 (76.1)	0.050

*SD* standard deviation

## Discussion

Because epidemiological data regarding MSK pain and rheumatic diseases in indigenous populations are scarce, we consider that the information yielded by this research is valuable. Our study is one of the first studies conducted among the indigenous population of Mexico. The study was carried out in two communities in the state of Oaxaca, revealing that MSK pain was present in 45.5 % of respondents, of whom 53.2 % had not received medical attention. It is striking that MSK pain and rheumatic diseases are highly prevalent in indigenous populations but are not treated in a timely fashion. The healthcare disadvantages in a rural setting, and especially the challenges to receiving health care faced by the indigenous populations of Latin America, have been documented [19] for a variety of acute and chronic conditions. However, the impact of chronic MSK disorders has not been detailed.

In our study, the most frequent diagnoses were mechanical, OA, and rheumatic regional pain syndrome. There were differences between the two populations. Whereas mechanical back pain predominated in the Mixtec group with 16.7 % versus 10.4 % in the Chontal group, OA was higher in the Chontal group (32.2 vs. 12.5 %). The prevalence of inflammatory back pain (4.1 %) was higher than the study previous done in Mexico (3 %). [8].

The prevalence in both groups is very high compared with other studies of indigenous populations, such as Aboriginal Australians and the Kaqchiquel of Guatemala, in whom back pain and OA are reported to have a very low prevalence compared with both communities in our study: 3.0 and 0.5 % for back pain and 4.3 and 1.6 % for OA, respectively [15, 16]. This variability may be potentially explained by the type of activities the population engages in. For example, men and women from the Chontal community are fully dedicated to agriculture, which requires them to do a great deal of walking on foot in a mountainous landscape, a highly taxing physical activity. Another possible explanation for this variability may lie in genetic aspects. Genetic studies were conducted as part of this study. The results will be reported in other publications and may partly shed light on these differences.

The prevalence of inflammatory rheumatic diseases such as RA, SLE, AS, and dermatomyositis was similar to that reported for other indigenous groups [15, 16] and the Mestizo population in Mexico [8]. These rheumatic diseases were found in the Mixtec community but not in the Chontal community, which may likely be explained by the smaller sample size of the latter.

The factors we found to be associated with diagnosis of a rheumatic disease were older age and lower education level. This is consistent with other surveys conducted using the COPCORD methodology, both nationwide [8–12] and in indigenous populations [15, 16]. This holds true even when the methodologies were different, such as a study of the native population of Manitoba, Canada, in which cases were identified using national registers [13]. As for sex, a greater proportion of women were affected. Although this difference was significant in our survey, the results are consistent with other reports, because most rheumatic diseases are more prevalent in women.

With regard to the presence of MSK pain, the rates reported in this survey are higher than in other COPCORD studies conducted in Mexico. A 2002 study [10] describing validation of the questionnaire for Mexico found a prevalence of 23 % in a suburban area of Mexico City. A later nationwide study in 2011 reported an average prevalence of 25.5 % in five different socioeconomic regions, albeit with significant variations across the regions (7.1–43.5 %) [8]. These variations led to the identification of two features present in populations with the highest prevalence rates: a high degree of marginalization characterized by poverty and insecurity (found in a sample from Mexico City), and living in a rural setting (identified in a rural area of the state of Nuevo León) [8]. These two characteristics are shared by the indigenous communities in our study.

The presence of MSK pain was also associated with other conditions, the most common and significant being high blood pressure, gastritis, anxiety, and depression. These results are in line with other COPCORD studies associating these comorbidities with MSK pain [8–12, 15, 16]. Our findings are also consistent with the COPCORD surveys conducted in Aboriginal Australians and Guatemala, both reporting high blood pressure and obesity as major comorbidities [15, 16]. The importance of this association lies in being able to treat potentially modifiable comorbidities such as obesity, smoking, and stress because complete elimination of or control over comorbidities would lessen symptoms of the disorder, especially the severity of MSK pain, and hence help to further reduce the potential for disability of these conditions.

The most frequently reported pain site among both Mixtec and Chontal populations was the back (15.4 %), which was similar to the figure reported for Aboriginal Australians, with lower back prevalence of 12.5 % [16]. In Guatemala, the most common site was the knee [15]. More often than not, pain in both sites was owing to osteoarthritis, which explains the MSK pain. Whereas the original COPCORD questionnaire relies on a visual analog scale to measure pain intensity, during the course of validating the translation we decided to use a Likert scale to assess pain intensity. On this scale, 32.2 % of participants reported having mild pain and 29.8 % reported the most severe pain. Despite this effort to adapt the methodology, we have yet to determine the perception and conception of pain intensity among these indigenous participants because it was not possible to discriminate between intensity and severity of pain owing to a language barrier. One of the research lines of the GLADERPO group is to qualitatively correlate pain measured using visual or Likert scales with the perception of pain, which changes depending on psychological, social, and cultural factors.

One of the hypotheses informing this research was that belonging to the indigenous population is a risk factor on its own for having rheumatic disease. However, our analysis revealed that speaking an indigenous language is also a borderline risk factor [32]. Although these results are not conclusive, we deem it justifiable to continue studies so as to properly assess indigenous populations in this regard. More robust data are needed to demonstrate whether indigenous populations possess inherent factors, such as genetics, as well as psychosocial and cultural aspects, and experience certain political and economic conditions that would render them more vulnerable to rheumatic disorders. These considerations are related to the chief strength of our study, having been geared specifically towards indigenous populations. The questionnaire has been validated cross-culturally for use in these communities and demonstrated sensitivity of 73.8 % and specificity of 72.9 % compared with a rheumatologist’s diagnosis [5, 18]. We also included variables such as self-identification as indigenous, speaking an indigenous language, and benefitting from a government program. A key question was whether participants considered themselves indigenous; the questionnaire was administered to those who answered positively to this question. Whereas 70.6 % of respondents were speakers of the Mixtec language, 100 % self-identified as indigenous. These aspects of defining the indigenous condition are being discussed in surveys being conducted worldwide [33].

Self-identification as indigenous can be defined from several standpoints, from the colonialist approach, to what is being contemplated by the UN Permanent Forum on Indigenous Issues, which has not yet come up with a universal definition of *indigenous*. A common approach recognizes a people as indigenous based on the sole fact that they inhabited the continent prior to the arrival of European conquerors, which thus fails to consider the enormous cultural, political, spiritual, ecological, and linguistic diversity of indigenous groups. The latter is the most important feature of self-identification and identification as indigenous [33]. In Mexico, the language criterion has been used in many censuses to define *indigenous*. Having included these considerations in our study has helped to ensure a response rate of 100 %. Such a high response rate contrasts with those from past surveys with lower response rates, especially in urban areas [8].

### Study limitations

The limitations of this study are those normally found in any cross-sectional study, i.e., that causality cannot be inferred from the results. The limited sample size of the Chontal indigenous group prevented determination of the actual prevalence of rheumatic diseases in this group, because we were unable to identify diseases such as AS, SLE, and others in the sample.

The prevalence of MSK disorders in indigenous communities of the Mixtec and Chontal regions of Oaxaca, Mexico was 45.5 %. The most common rheumatic diseases were back pain and osteoarthritis.

Given the disabilities produced by these disorders and the high prevalence revealed in this survey, we believe that these results should be considered when planning health care programs. Rheumatic diseases are currently not included in the universal healthcare insurance proposed by the Mexican government. These findings may also prove useful to encourage the inclusion of early identification and appropriate treatment of these highly prevalent disorders in medical training programs and medical practice at large. Finally, these results lay the groundwork for phase II of the COPCORD methodology, which consists of proposing diagnosis and treatment programs consistent with epidemiological and sociocultural characteristics of the target population.
